# Conversion of viridicatic acid to crustosic acid by cytochrome P450 enzyme-catalysed hydroxylation and spontaneous cyclisation

**DOI:** 10.1007/s00253-021-11674-4

**Published:** 2021-11-11

**Authors:** Jenny Zhou, Shu-Ming Li

**Affiliations:** Institut Für Pharmazeutische Biologie Und Biotechnologie, Fachbereich Pharmazie, Philipps-Universität Marburg, Robert-Koch-Straße 4, 35037 Marburg, Germany

**Keywords:** Cytochrome P450, Hydroxylation, Terrestric acid biosynthesis, Split marker approach, Marker recycling

## Abstract

**Abstract:**

Cytochrome P450 monooxygenases (P450s) are considered nature’s most versatile catalysts and play a crucial role in regio- and stereoselective oxidation reactions on a broad range of organic molecules. The oxyfunctionalisation of unactivated carbon-hydrogen (C-H) bonds, in particular, represents a key step in the biosynthesis of many natural products as it provides substrates with increased reactivity for tailoring reactions. In this study, we investigated the function of the P450 enzyme TraB in the terrestric acid biosynthetic pathway. We firstly deleted the gene coding for the DNA repair subunit protein Ku70 by using split marker-based deletion plasmids for convenient recycling of the selection marker to improve gene targeting in *Penicillium crustosum*. Hereby, we reduced ectopic DNA integration and facilitated genetic manipulation in *P. crustosum*. Afterward, gene deletion in the Δ*ku70* mutant of the native producer *P. crustosum* and heterologous expression in *Aspergillus nidulans* with precursor feeding proved the involvement of TraB in the formation of crustosic acid by catalysing the essential hydroxylation reaction of viridicatic acid.

**Key points:**

*•Deletion of Ku70 by using split marker approach for selection marker recycling.*

*•Functional identification of the cytochrome P450 enzyme TraB.*

*•Fulfilling the reaction steps in the terrestric acid biosynthesis.*

**Supplementary Information:**

The online version contains supplementary material available at 10.1007/s00253-021-11674-4.

## Introduction

Natural products (NPs) isolated from microorganisms are well-known for their biological activities and are promising candidates for drug development (Katz and Baltz 2016). Fungi, especially those isolated from the marine environment, are reported to produce many NPs that exhibit anti-bacterial, anti-fungal, anti-oxidant, anti-inflammatory, and even cytotoxic activities and have therefore largely contributed to the expansion of the pharmaceutical repertoire (Keller 2019; Shabana et al. 2021). Recent advances in targeted gene manipulation, including gene disruption and heterologous expression, allowed the analysis of gene functions and the elucidation of NP biosynthetic pathways (Atanasov et al. 2021).

Cytochrome P450 enzymes (P450s) are a ubiquitously occurring superfamily of heme-thiolate proteins and are distributed in all kingdoms of life, including humans, animals, plants, fungi, and bacteria (Harken and Li 2021; McIntosh et al. 2014; Zhang et al. 2021). They catalyse oxidation reactions in the biosynthesis of many natural products, such as alkaloids, terpenes, and polyketides. These reactions are involved in the functionalisation of non-reactive carbons by aliphatic and aromatic bond hydroxylation, epoxidation, heterocyclisation, ring-coupling, and C–C bond cleavage and are often performed with remarkable regio- and stereoselectivity (Podust and Sherman 2012).

**Fig. 1:**
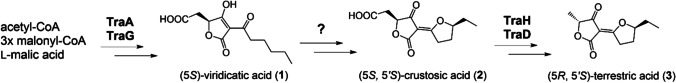
Proposed biosynthetic pathway of terrestric acid in *P. crustosum* PRB-2 modified after Fan et al. (2020)

Our group recently identified a tetronic acid biosynthetic gene cluster (*pcr11009-pcr11016*, *traA-H*) in *Penicillium crustosum* PRB-2 (Fan et al. 2019, 2020). Biochemical investigation proved the function of the hybrid PKS-NRPS TraA and the enoyl reductase TraG for the formation of viridicatic acid (**1**), which is subsequently hydroxylated and undergoes ring formation to yield crustosic acid (**2**). The nonheme Fe^II^-2OG-dependent oxygenase TraH catalyses an oxidative decarboxylation of **2**, followed by stereospecific reduction with the flavin-containing oxidoreductase TraD to form the final product terrestric acid (**3**) (Fan et al. 2019, 2020) (Fig. 1). TraB from this cluster was proposed as the responsible enzyme for the hydroxylation reaction of **1**. To verify TraB function, Fan et al. performed gene deletion using the *hph* gene as selective marker (Fan et al. 2019). However, deletion of *traB* in *P. crustosum* did not lead to the accumulation of **1**. Instead, accumulation of clavatol, hydroxyclavatol, and hydroxyclavatol methyl ether was detected, which resembled the secondary metabolite profile of the Δ*traA* deletion mutant reported in the same study. A possible reason for these unexpected results could be the unintentional polar effect on *traA*. In this study, we repeated the *traB* deletion in *P. crustosum* by preparing a new deletion cassette and proved its function as the proposed hydroxylase.

## Materials and methods

### Strains, media, and culture conditions

Fungal strains used and created in this study are listed in Table S1 (Supplementary Information). *Saccharomyces cerevisiae* strain HOD114-2B was used for cloning via homologous recombination. The yeast was grown at 30 °C in YPD medium (1% yeast extract, 2% peptone, and 2% glucose). For selection of transformants, synthetic complete medium without uracil (6.7 g/L yeast nitrogen base with ammonium sulphate, 650 mg/L CSM-His-Leu-Ura, 20 mg/L histidine, and 60 mg/L leucine) was used.

*Escherichia coli* strain DH5α was used for plasmid propagation and cultivated at 37 °C in LB medium (lysogeny broth). For selection of recombinant strains, ampicillin (50 mg/L) was added to the medium.

*Penicillium crustosum* strain PRB-2 (Wu et al. 2012; Yu et al. 2019) was deposited as strain MFCSCS 200 in the culture collection of the Key Laboratory of Marine Drugs, the Ministry of Education of China, School of Medicine and Pharmacy, Ocean University of China, Qingdao, People’s Republic of China. All *pyrG* intact strains were cultivated standing at 25 °C in PDB medium (24 g/L potato dextrose broth, Sigma-Aldrich). *pyrG* deficient strains were cultivated on PDB medium supplemented with 0.5 g/L uridine and 0.5 g/L uracil. For *pyrG* marker recycling, 7.5 mM 5-fluoroorotic acid (5-FOA), 0.5 g/L uridine, and 0.5 g/L uracil were added to the medium at pH 4.

*Aspergillus nidulans* strains were cultivated standing at 37 °C in PDB medium supplemented with 0.5 g/L uridine, 0.5 g/L uracil, 2.5 mg/L riboflavin, and/or 0.5 mg/L pyridoxine, respectively, depending on the used selection markers.

### Isolation of fungal genomic DNA

For isolation of genomic DNA, *P. crustosum* and *A. nidulans* strains were cultivated in 500 μL LMM medium (1.0% glucose, 50 mL/L salt solution, 1 mL/L trace element solution, and 0.5% yeast extract) with appropriate supplementation for 3 days at 30 °C. The mycelia were then collected by centrifugation at 13,300 rpm for 10 min. Four hundred microlitres of LETS-buffer (10 mM Tris–HCl pH 8.0, 20 mM EDTA, 0,5% SDS, 0.1 M LiCl) were added, and the mycelia were crushed with glass beads in a MiniLys homogeniser (Bertin Technologies, Montigny-le-Bretonneux. France) for 3 min at full speed. After adding 300 μL LETS-buffer, the solution was treated with 700 μL phenol/chloroform/isoamyl alcohol (25:24:1), mixed by inverting and centrifuged at 13,000 rpm for 10 min. Subsequently, the aqueous phase was treated with 900 μL absolute ethanol for DNA precipitation and centrifuged at 13,300 rpm, 4 °C for 30 min. The DNA was washed with 500 μL 70% ethanol, dried, and dissolved in 50 μL distillated H_2_O.

### Genome sequencing and sequence analysis

Genome sequencing was performed by BerryGenomics (Beijing, China) using Novaseq6000/X-ten (Illumina). The prediction of open reading frames was performed using secondFind (http://biosyn.nih.go.jp/2ndfind/). Enzyme functions were predicted with online BLAST approaches (http://blast.ncbi.nlm.nih.gov). The nucleotide sequence of the terrestric acid cluster reported in this study is available at GenBank under accession number MK360919 and that of Ku70 under KAF7525404.1.

### PCR amplification, gene cloning, and plasmid construction

Plasmids generated and used in this study are listed in Table S2. The primers used for PCR amplification were synthesised by Seqlab GmbH (Göttingen, Germany) and are listed in Table S3. For PCR amplification, Phusion High-Fidelity DNA polymerase from New England Biolabs (NEB) and a T100TM Thermal cycler from Bio-Rad were used.

Plasmids for gene deletion and heterologous expression were constructed by homologous recombination in *Escherichia coli* or *Saccharomyces cerevisiae*. For gene deletion experiments followed by subsequent marker recycling, a deletion cassette was prepared using two split marker constructs. One of them contained the approximately 1.5 kbps upstream DNA sequence of the target gene, 300 bps from the downstream region for subsequent marker recycling, and two-thirds of the *afpyrG* selection marker with 25–30 bps overlap to the *Sma*I restriction site of the pESC-URA vector. The second construct consists of another two-thirds of the *afpyrG* cassette, the approximately 1.5 kbps downstream DNA sequence of the target gene, and was also introduced into the *Sma*I restriction site of linearised vector pESC-URA. The *afpyrG* resistance cassette originated from pFK23 (Kindinger et al. 2019).

The plasmid for heterologous expression of *traB* in *A. nidulans* was constructed by PCR amplification of *traB* including its terminator of 632 bp from genomic DNA of *P. crustosum* and subsequent cloning into *Sfo*I*-*linearised vector pJN017 (Kindinger et al. 2019) containing the flanking regions of the *wA* gene, the *gpdA* promoter, and the *afribo* selection marker. The amplified genomic sequence of *traB* was cloned between the *gpdA* promoter and the *afribo* marker by using 25–30 bps overlap to the *Sfo*I restriction site.

### Genetic manipulation in *P. crustosum* FK15, JZ02, and *A. nidulans* LO8030

Transformation of *P. crustosum* FK15 (Kindinger et al. 2019), JZ02, and *A. nidulans* LO8030 (Chiang et al. 2016) was performed by polyethylene glycol (PEG)–mediated protoplast transformation. Fresh spores of FK15 or JZ02, respectively, were inoculated in a 250-mL flask containing 30 mL LMM medium (1.0% glucose, 50 mL/L salt solution, 1 mL/L trace element solution, and 0.5% yeast extract) supplemented with 0.5 g/L uridine and 0.5 g/L uracil and incubated at 25 °C, 230 rpm for 11 h. Spores of LO8030 were inoculated in a 250-mL flask containing 30 mL LMM medium supplemented with 0.5 g/L uridine, 0.5 g/L uracil, 2.5 mg/L riboflavin, and 0.5 mg/L pyridoxine and incubated at 37 °C, 230 rpm for 5 h. The germlings were collected by centrifugation at 5000 rpm for 5 min and washed with 30 mL distillated H_2_O and 10 mL osmotic buffer (1.2 M MgSO_4_ in 10 mM sodium phosphate, pH 5.8). For cell wall degradation, the germlings were transferred into a 100-mL flask containing 10 mL osmotic buffer, 50 mg lysing enzyme from *Trichoderma harzianum* (Sigma), and 20 mg yatalase from *Corynebacterium* sp. OZ-21 (OZEKI Co., Ltd.) and incubated at 30 °C, 100 rpm. After 3 h, the protoplasts were transferred into a 50-mL falcon tube, overlaid with trapping buffer (0.6 M sorbitol in 0.1 M Tris-HCI, pH 7.0), and centrifuged at 5000 rpm for 15 min at 4 °C. The cells were then collected from the interphase and carefully transferred into a 15-mL falcon tube. After centrifugation at 5000 rpm for 7 min, the pellet was resuspended in 200 μL cold STC buffer (1.2 M sorbitol, 10 mM CaCl_2_, and 10 mM Tris-HCI, pH 7.5). For transformation, the protoplasts were treated with 3 μg DNA and incubated on ice for 50 min. Then, 1.25 mL PEG solution (60% PEG 4000, 50 mM CaCl_2_, 50 mM Tris-HCI, pH 7.5) was added, gently mixed, and incubated at room temperature for 25 min. Five millilitres of STC buffer was added to the mixture, which was then transferred onto plates with SMM bottom medium (1.0% glucose, 50 mL/L salt solution, 1 mL/L trace element solution, 1.2 M sorbitol, 1.6% agar). The plates were overlaid with SMM top medium (1.0% glucose, 50 mL/L salt solution, 1 mL/L trace element solution, 1.2 M sorbitol, and 0.8% agar) and incubated at 25 °C for 5 days for *P. crustosum* or 37 °C for 3 days for * A. nidulans*. For *A. nidulans* LO8030, the SMM bottom and top media were additionally supplemented with 0.5 g/L uridine, 0.5 g/L uracil, and 0.5 mg/L pyridoxine. The transformants were transferred onto fresh GMM plates (1.0% glucose, 50 mL/L salt solution, 1 mL/L trace element solution, and 1.6% agar) with appropriate supplementation and, subsequently, inoculated for the isolation of genomic DNA to verify positive transformants via PCR.

### PCR verification of positive transformants and cultivation for secondary metabolite production

Genomic DNA was isolated from the transformants and used for PCR amplification. PCR for the control of gene deletion transformants was performed using one primer binding outside of the deletion cassette and the second one binding in the marker gene. To confirm deletion of the *afpyrG* gene by marker recycling, primers were designed to amplify the flanking regions with or without the *pyrG* sequence to determine if the selection marker is present. For *traB* overexpression strains, primers binding outside of the expression construct and the counterpart binding in the *afribo* marker or the *gpdA* promoter, respectively, were used. In addition, the presence of *traB* was proven by PCR amplification of the expressed gene. The PCR results are given in Figs. S1, S2, S3, and S4.

For detection of secondary metabolites, transformants were cultivated in PDB medium with the required supplements. After 14 days, secondary metabolites were extracted with equivalent volumes of ethyl acetate for three times, dissolved in a mixture of methanol and distilled H_2_O (9:1) and analysed via LC–MS.

### Precursor feeding in *A. nidulanstraB* expression strain

Five millilitres of PDB medium supplemented with 0.5 g/L uridine, 0.5 g/L uracil, and 0.5 mg/L pyridoxine was inoculated with spores of *A. nidulans traB* expression strain JZ01 or empty vector control strain BK06, respectively. Substrate **1** was dissolved in DMSO to give a 200 mM stock solution and fed to the PDB cultures directly after inoculation in an appropriate amount to a final concentration of 0.2 mM. The cultures were incubated standing at 25 °C for 7 days. Secondary metabolites were then extracted with equivalent volumes of ethyl acetate for three times, dissolved in a mixture of methanol and distilled H_2_O (9:1), and analysed via LC–MS.

### Large-scale fermentation, extraction, and isolation of secondary metabolites

To isolate **1**, spores of the *P. crustosum* Δ*traB* mutant strain were inoculated into 20 × 250 mL Erlenmeyer flasks containing 100 mL PDB liquid medium each and cultivated at 25 °C under static conditions for 14 days. After separating mycelia by filtration, the supernatant was extracted with equal volumes of ethyl acetate for three times and evaporated under reduced pressure to give a crude extract (1.2 g). For purification, the crude extract was subjected to silica gel column chromatography and eluted with a gradient of MeOH/CH_2_Cl_2_ (9:1 to 1:1) to give 16 fractions. Fraction 12–14 were pooled and evaporated under reduced pressure to obtain **1** (293 mg).

To isolate **2**, spores of *A. nidulans traB* expression strain JZ01 were inoculated into two 250-mL Erlenmeyer flasks containing 100 mL PDB liquid medium each, supplemented with 0.5 g/L uracil, 0.5 g/L uridine, and 0.5 mg/L pyridoxine. Thirty milligrammes of substrate **1** isolated from the *P. crustosum* Δ*traB* mutant strain was dissolved in DMSO, fed to the PDB cultures, and cultivated on a rotary shaker at 200 rpm and 25 °C for 8 days. After separating mycelia by filtration, the supernatant was extracted with equal volumes of ethyl acetate for three times and evaporated under reduced pressure to give a crude extract (70 mg). For purification, the crude extract was subjected to silica gel column chromatography and eluted with a gradient of MeOH/CH_2_Cl_2_ (9:1 to 1:1) to give 40 fractions. The pooled fractions 28–34 were further purified on a semi-preparative HPLC (H_2_O/CH_3_CN (90:10 to 0:100 in 15 min) to yield **2** (1 mg).

### LC–MS analysis of secondary metabolites

For the analysis of secondary metabolites via LC–MS, an Agilent 1260 series HPLC equipped with a Multospher 120 RP 18-5μ column (250 × 2 mm, CS-Chromatographie Service GmbH) was used. The mobile phase consists of solvent A (H_2_O with 0.1% HCOOH) and solvent B (CH_3_CN with 0.1% HCOOH) at a flow rate of 0.25 mL/min. The substances were eluted using a linear gradient from 5 to 100% B within 40 min. For mass determination, positive ion mode electrospray ionisation (ESI) in a micrOTOF-Q III mass spectrometer with ESI-source (Bruker Daltonics) was used with 5 mM sodium formiate for mass calibration. LC–MS data were evaluated with DataAnalysis 4.2 software (Bruker Daltonik, Bremen, Germany).

### NMR analysis

Isolated compounds were dissolved in DMSO-d_6_ for structure elucidation via ^1^H-NMR analysis. NMR spectra (Figs. S5 and S6) were taken on a JEOL ECA-500 MHz spectrometer (JEOL, Tokyo, Japan) and processed using MestReNova 6.1.0 (Mestrelab). All chemical shifts (Table S4) are referenced to the shift of the solvent signal.

## Results

### Construction of *P. crustosumku70* (*pcr4870*) deletion strain using split marker approach

To improve gene-targeting efficiency in *Penicillium crustosum*, we aimed to identify and delete the gene coding for the DNA repair protein subunit Ku70. Ku70 as part of the Ku70/Ku80 heterodimer is involved in the recognition of DNA double-strand breaks and increases non-homologous DNA end-joining events (Pannunzio et al. 2018). This DNA repair mechanism prohibits homologous recombination events in *P. crustosum*. Deletion of *ku70* has been proven to increase the gene-targeting frequency in many fungal species including *Penicillium marneffei* (Bugeja et al. 2012) and *Penicillium digitatum* (Gandía et al. 2016).

To find a Ku70 candidate, the putative Ku70 homologue CRL31047.1 from *Penicillium camemberti* was used for BLAST search, leading to the identification of KAF7525404.1 comprising 653 residues from *P. crustosum* strain G10 with a sequence identity of 98.6% on the amino acid level. An orthologue Pcr4870 was identified in the genome sequence of *P. crustosum* PRB-2 and differs from KAF7525404.1 at only two residues. Proline at position 43 and threonine at 454 in KAF7525404.1 were replaced by serine and isoleucine in Pcr4870, respectively. Nucleotide sequence comparison revealed differences at four positions. C182, C1463, G1836, and G2071 in the gene coding for KAF7525404.1 were replaced by T182, T1463, A1836, and A2071 in *pcr4870* in strain PRB-2, respectively.

**Fig. 2:**
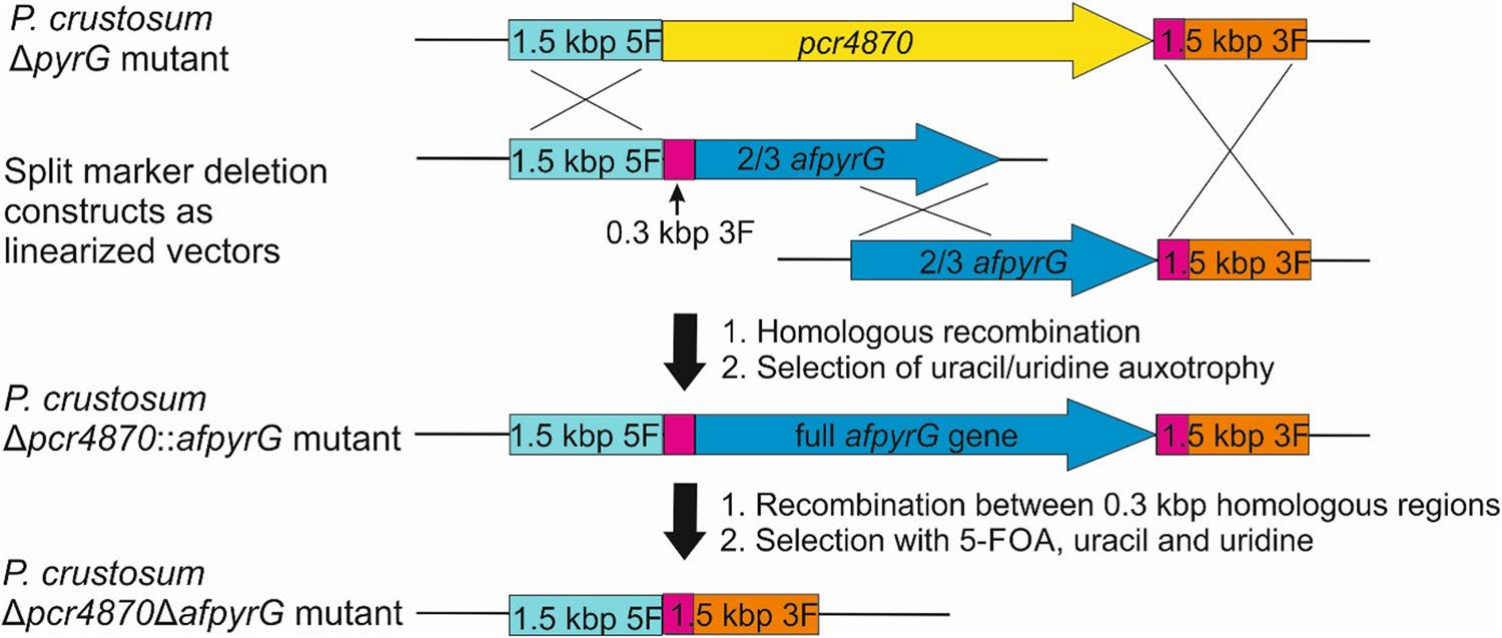
Strategy for deletion of *pcr4870* in *P. crustosum* using split marker approach followed by selection marker recycling

Taking into consideration to use the Δ*pcr4870* strain for future gene deletion experiments due to its improved gene targeting, we designed deletion constructs according to the protocol of Maruyama and Kitamoto (2008) to allow multiple gene disruptions by using selection marker recycling. For the deletion of *pcr4870*, a plasmid was designed containing the 1.5 kbp flanking regions of *pcr4870* and the *afpyrG* selection marker originated from *Aspergillus fumigatus*. In addition, a 0.3 kbp fragment of the downstream region is cloned between the upstream sequence and the *afpyrG* marker to allow subsequent marker recycling as reported by Maruyama and Kitamoto. However, construction of the desired plasmid in both yeast and *E. coli* was unsuccessful. After closely reviewing the cloning strategy described by Maruyama and Kitamoto, it became obvious that inevitable recombination between the 0.3 kbp homologous sequences of the downstream region led to the excision of the *afpyrG* marker during the cloning procedure. To avoid having the 0.3 kbp homologous regions in one construct, we designed new plasmids using the split marker approach in which each plasmid contains two-thirds of the *afpyrG* marker, next to the up- or downstream sequences of *pcr4870*, respectively. The 0.3 kbp fragment of the downstream region is cloned between the upstream sequence and the selection marker to allow subsequent marker recycling by homologous recombination as shown in Fig. 2.

The linearised DNA fragments for disruption of the *pcr4870* gene were introduced into the *P. crustosum* Δ*pyrG* strain FK15 and selection based on uracil and uridine auxotrophy resulted in Δ*pcr4870*::*afpyrG* strain JZ02p. Subsequently, selection on plates supplemented with 5-FOA, uracil, and uridine led to the excision of the *afpyrG* marker by recombination between the 0.3 kbp homologous regions before and after the *afpyrG* sequence to yield Δ*pcr4870*Δ*afpyrG* strain JZ02. Transformants were confirmed by PCR verification as shown in Figs. S1 and S2.

### Inactivation of *traB* in *P. crustosum* led to the accumulation of viridicatic acid

**Fig. 3:**
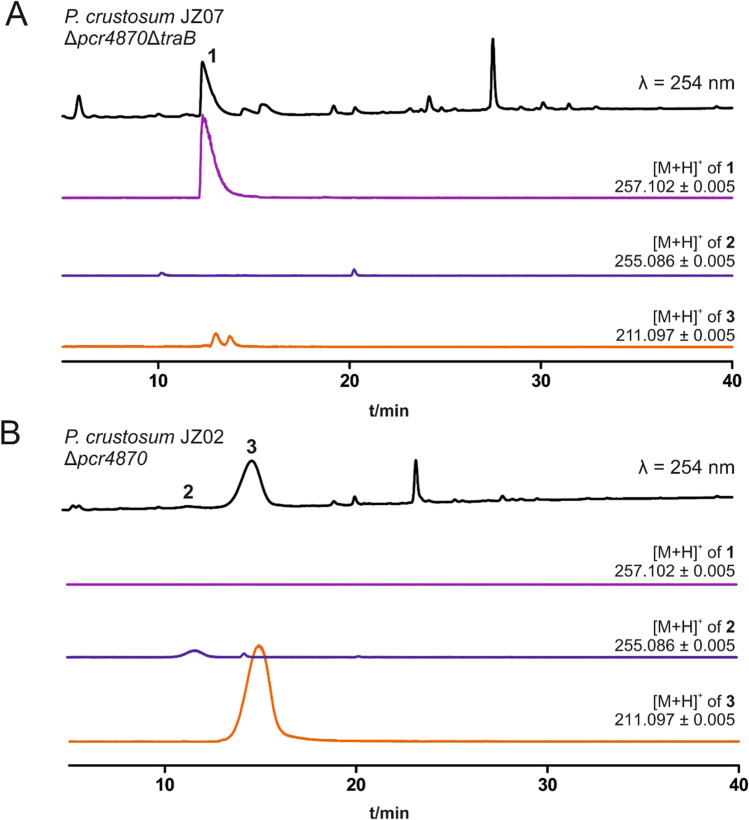
LC–MS detection of secondary metabolites from *P. crustosum* strains. 14-day liquid PDB surface culture of Δ*traB*Δ*pcr4870* strain JZ07 (**A**) and Δ*pcr4870* strain JZ02 (**B**)

As aforementioned, the putative cytochrome P450 enzyme TraB (Pcr11010) in *P. crustosum* was speculated to be responsible for the formation of **2** in the biosynthesis of **3** (Fig. 1) (Fan et al. 2019). However, this hypothesis was not proven experimentally. We therefore decided to repeat the gene deletion experiment. To maintain the option of further gene deletions in the *traB* disruption strain using the same selection marker, we designed split marker constructs for subsequent marker recycling as described above. The 1.5 kbp upstream flanking region of *traB* and 300 bp from the downstream sequence were amplified from genomic DNA of *P. crustosum* wild-type strain PRB-2, fused with two-thirds of the *afpyrG* marker and cloned into linearised vector pESC-URA. The second split marker plasmid contained 1.5 kbp of the downstream flanking region and another two-thirds of the *afpyrG* selection marker. Both DNA fragments for deletion of *traB* were linearised and introduced into the *P. crustosum pcr4870* disruption strain JZ02, resulting in Δ*pcr4870*Δ*traB*::*afpyrG* strain JZ07. Transformants were selected based on uracil/uridine auxotrophy and additionally verified by PCR (Fig. S3). Cultivation of JZ07 in rice and PDB medium revealed that the inactivation of TraB almost completely abolished the production of **2** and **3** when compared to the secondary metabolite profile of Δ*pcr4870*Δ*afpyrG* strain JZ02. Instead, clear accumulation of **1** could be detected (Fig. 3). Structural elucidation via LC–MS and ^1^H-NMR analyses (Fig. S5, Table S4) confirmed **1** to be (*5S*)-viridicatic acid (Fan et al. 2020).

### Heterologous expression of *traB* in *A*. *nidulans* and feeding of viridicatic acid led to crustosic acid formation

Having identified the involvement of TraB in the terrestric acid biosynthesis, we proceeded to investigate the conversion of **1** to **2** by heterologous expression of TraB in *Aspergillus nidulans* LO8030. For this purpose, the whole genomic sequence of *traB* including its native terminator was amplified from *P. crustosum* wild-type strain PRB-2 and cloned into the expression vector pJN017 under the control of the *gpdA* promoter. The expression construct was then linearised and site-specifically introduced into the *wA* locus of *A. nidulans* strain LO8030, resulting in *A. nidulans traB* expression strain JZ01. Transformants were selected based on riboflavin auxotrophy, and those showing an albino phenotype, indicating successful integration into the *wA* locus, were additionally verified via PCR (Fig. S4). In parallel, empty vector pJN017 was linearised and introduced into the *wA* locus of *A. nidulans* to give control strain BK06, which was kindly provided by Bastian Kemmerich from our group.

**Fig. 4:**
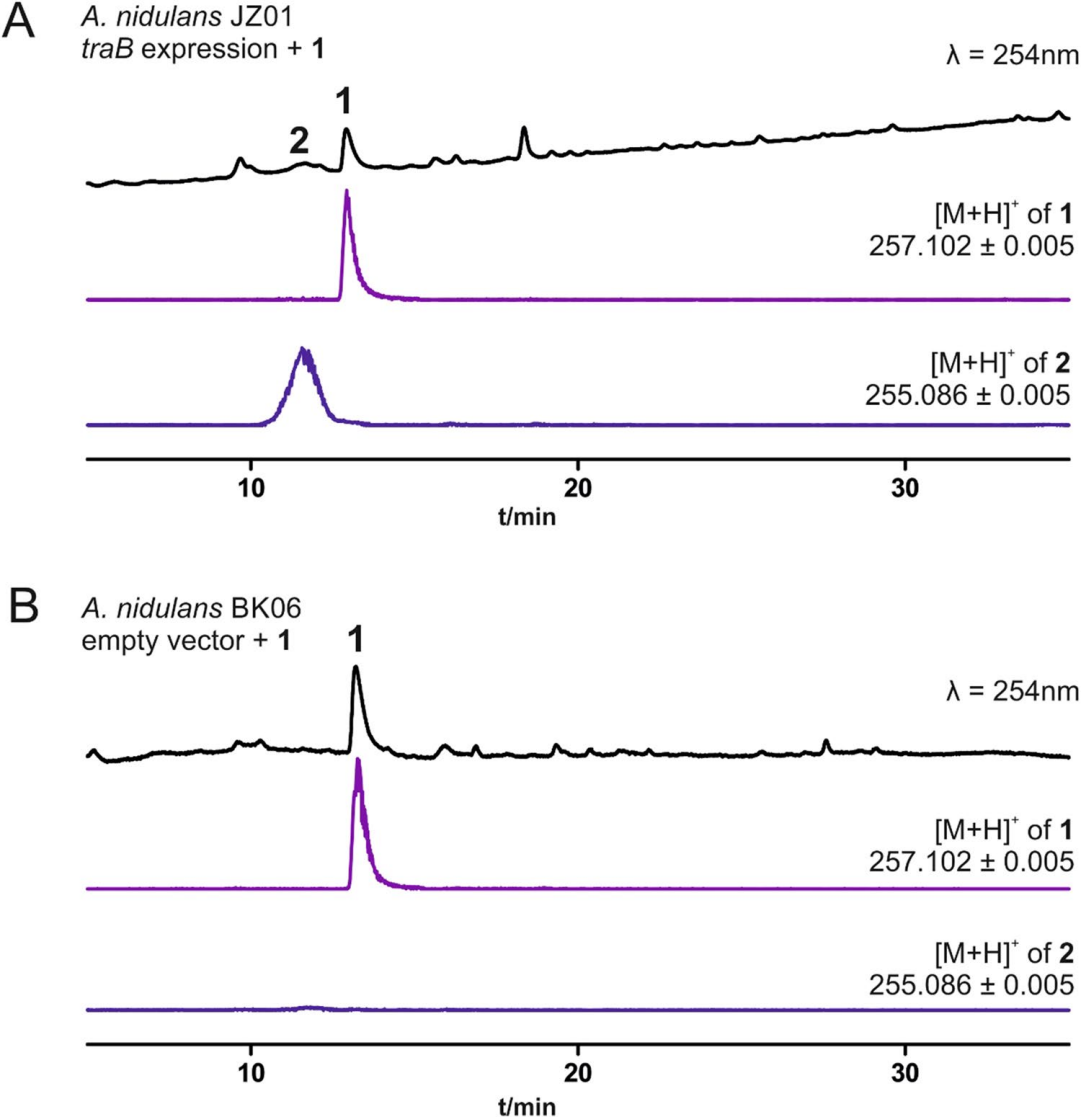
LC–MS detection of secondary metabolites from *A. nidulans* strains after feeding with 1. 7-day liquid PDB surface culture of *traB* overexpression strain JZ01 (**A**) and empty vector control BK06 (**B**)

To analyse the function of TraB, substrate **1** was dissolved in DMSO and fed to the cultures of both overexpression strain JZ01 and control strain BK06 in PDB medium directly after inoculation. After incubation for 7 days, secondary metabolites were extracted with ethyl acetate and analysed via LC–MS. As expected, feeding **1** in the *traB* overexpression strain led to the formation of **2**, which was not observed in empty vector control strain BK06 (Fig. 4). For structure confirmation, **2** was isolated from the *traB* overexpression strain JZ01 after feeding with **1** and analysed via LC–MS and ^1^H-NMR (Fig. S6, Table S4), proving it unequivocally as (*5S*, *5´S*)-crustosic acid (Fan et al. 2019).

## Discussion

Cytochrome P450 enzymes largely contribute to the high structural diversity of natural products as they catalyse the oxidation of C-H groups into more active functional groups (Crešnar and Petric 2011). These P450-mediated functionalisation reactions of unactivated carbon skeletons significantly increase the reactivity and are therefore often essential to provide substrates for tailoring reactions, demonstrating the role of P450s as key enzymes in many biosynthetic pathways (Münch et al. 2021).

In a previous study, our group has demonstrated that the hydroxylation reaction is crucial for the formation of **2**. By incubating **2** in D_2_O, it was observed that **2** is a closed, anhydrous form of a hydroxylated derivative of **1** (Fan et al. 2020). It is very likely that hydroxylated **1** can be converted to **2** by spontaneous ring formation. Our main object in this study was the identification of the enzyme responsible for the hydroxylation reaction in the aliphatic side chain of **1**, the last unknown step in the terrestric acid biosynthesis. As hydroxylation is typically catalysed by P450s, it was speculated that the P450 TraB as a member of the terrestric acid cluster is involved in this reaction.

To facilitate targeted genetic manipulation in *P. crustosum*, we first tried to delete the gene encoding for DNA repair subunit protein Ku70 using the marker recycling strategy according to the protocol of Maruyama and Kitamoto (2008). However, recombination between the 0.3 kbp homologous regions during the cloning procedure required application of the split marker approach to obtain the desired constructs in two plasmids. Our split marker approach for the construction of deletion plasmids with subsequent marker recycling is applicable for standard cloning strategies with strains like DH5α. In addition, using split marker plasmids for transformation enhances gene targeting due to the requirement of more homologous recombination events in comparison to using a single plasmid. In *Penicillium chrysogenum*, synergistic effects on gene-targeting efficiency have been reported for applying split marker constructs for the transformation of mutants with a Δ*ku70* or Δ*ligD* background (de Boer et al. 2010).

**Fig. 5:**
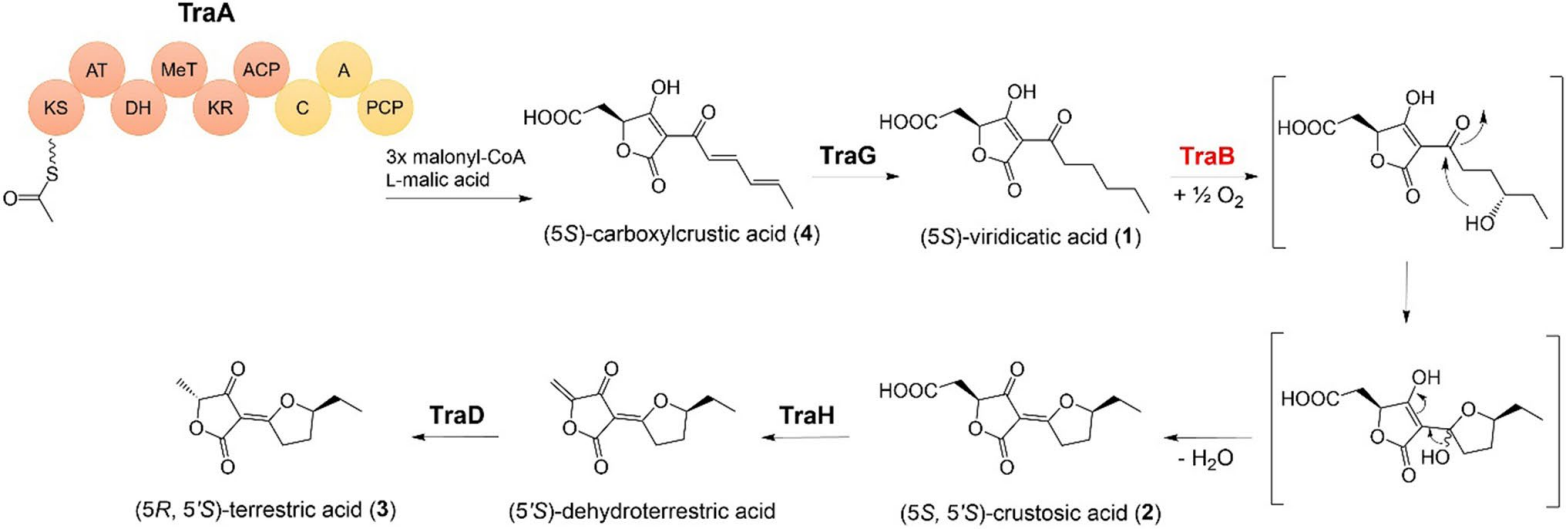
Biosynthetic pathway of terrestric acid in *P. crustosum* PRB-2

Our gene deletion experiment in native producer *P. crustosum* proved the role of TraB as the key enzyme for the hydroxylation of **1**, as accumulation of **1** was detected after deletion of *traB*. Heterologous expression of *traB* in host organism *A. nidulans* and feeding of the substrate **1** led to the formation of **2**, confirming that TraB alone is responsible for hydroxylation of **1**. Nucleophilic addition of the hydroxyl group to the carbonyl group at the side chain and subsequent dehydration result in **2** (Fig. 5).

Taken together, we optimised the marker recycling strategy by Maruyama and Kitamoto (2008), so that it can be applied for conventional cloning methods. In addition, we improved gene-targeting frequency by deletion of the gene for non-homologous end-joining events. We proved TraB as viridicatic acid hydroxylase by gene deletion in the producer *P. crustosum* and heterologous expression in *A. nidulans*. With this study, we completely elucidated the reaction steps in the biosynthesis of terrestric acid with their responsible enzymes (Fig. 5).

## Supplementary Information

Below is the link to the electronic supplementary material.

Supplementary file1 (PDF 956 KB)
