# Supplementary material for: Conversion of viridicatic acid to crustosic acid by cytochrome P450 enzyme-catalysed hydroxylation and spontaneous cyclisation
Source: Appl Microbiol Biotechnol. 2021 Nov 11;105(24):9181–9. doi: 10.1007/s00253-021-11674-4 (PMC8648635; doi:10.1007/s00253-021-11674-4)
Supplement: Supplementary file 1 — Supplementary file1 (PDF 956 KB) [file 253_2021_11674_MOESM1_ESM.pdf]

Supporting Information

Journal:

Applied Microbiology and Biotechnology

Title:

Conversion of viridicatic acid to crustosic acid by cytochrome P450 enzyme-catalysed hydroxylation and spontaneous cyclisation

Authors:

Jenny Zhou and Shu-Ming Li*

Affiliation:

Institut für Pharmazeutische Biologie und Biotechnologie, Fachbereich Pharmazie, Philipps-Universität Marburg, Robert-Koch-Straße 4, 35037 Marburg, Germany.

Corresponding Author:

*Tel/Fax: + 49-6421-28-22461/25365. E-mail: shuming.li@staff.uni-marburg.de.

ORCID Shu-Ming Li: 0000-0003-4583-2655

Table of contents

Table S1: Fungal strains used and generated in this study S3

Table S2: Plasmids used and generated in this study S4

Table S3: Primers used in this study........ S5

Table S4: ^{1}H NMR data of compound **1** and **2** S6

Fig. S1 PCR verification of Δ*pcr4870* mutant by amplification of two different fragments from genomic DNA of wild type and Δ*pcr4870* mutant (schematic representation)........ S7

Fig. S2 PCR verification of Δ*pcr4870*Δ*afpyrG* mutant. Amplification from two different fragments from Δ*pcr4870*::*afpyrG* and *Δpcr4870ΔafpyrG* strains proved the presence/absence of the *afpyrG* marker (schematic representation). S8

Fig. S3 PCR verification of Δ*traB* mutant by amplification of two different fragments from genomic DNA of wild type and Δ*traB* mutant (schematic representation)........ S9

Fig. S4 PCR verification of *traB* overexpression mutant by amplification of three different fragments including the *traB* gene itself from genomic DNA of *A. nidulans* LO8030 and *traB* expression strain JZ01 (schematic representation)........ S10

Fig. S5 ^{1}H NMR spectrum of compound **1** in DMSO-d_6 (400 MHz) S11

Fig. S6 ^{1}H NMR spectrum of compound **2** in DMSO-d_6 (400 MHz) S12

References........ S13

Table S1: Fungal strains used and generated in this study

Strain	Genotype	Source
Penicillium crustosum		
PRB-2	wild type	(Wu et al. 2012)
FK15	Δ*pyrG* in *P. crustosum* PRB-2	(Kindinger et al. 2019)
JZ02p	Δ*pcr4870*::*afpyrG* in *P. crustosum* FK15	This study
JZ02	Δ*pcr4870*Δ*afpyrG* in *P. crustosum* JZ02p	This study
JZ07	Δ*pcr4870*Δ*traB*::*afpyrG* in *P. crustosum* JZ02	This study
Aspergillus nidulans		
LO8030	*pyroA4, riboB2, pyrG89, nkuA::argB*, sterigmatocystin cluster (AN7804-AN7825)Δ, emericellamide cluster (AN2545-AN2549)Δ, asperfuranone cluster (AN1039-AN1029)Δ, monodictyphenone cluster (AN10023-AN10021)Δ, terrequinone cluster (AN8512-AN8520)Δ, austinol cluster part 1 (AN8379-AN8384)Δ, austinol cluster part 2 (AN9246-AN9259)Δ, F9775 cluster (AN7906-AN7915)Δ, asperthecin cluster (AN6000-AN6002)Δ	(Chiang et al. 2016)
BK06	Δ*wA-PKS*::*gpdA*::*afribo* in *A. nidulans* LO8030	This study
JZ01	Δ*wA-PKS*::*gpdA*::*traB*::*afribo* in *A. nidulans* LO8030	This study

Table S2: Plasmids used and generated in this study

Plasmid	Description	Source
pESC-URA	*Saccharomyces cerevisiae* and *E. coli* shuttle vector	Agilent
pFK23	*URA3*, *pcr4401* flanking, *A. nidulans gpdA* promoter, *A. fumigatus pyrG* (*afpyrG*), *ampR*	(Kindinger et al. 2019)
pJN017	*URA3*, *wA* flanking, *A. nidulans gpdA* promoter, *A. fumigatus riboB* (*afribo*), *ampR*	(Kindinger et al. 2019)
pJZ02	two-thirds of the *afpyrG* marker at the 3'-end (1110 bps) originated from pFK23 was fused to the 1495 bps PCR fragment of the downstream region from *pcr4870* from genomic DNA of *P. crustosum* PRB-2 and inserted into the pESC-URA vector	This study
pJZ04	a 1494 bps PCR fragment of the upstream region from *pcr4870* from genomic DNA of *P. crustosum* PRB-2 was fused to 297 bps of the downstream region, a 1843 bps PCR fragment including the *gpdA* promoter and two-thirds of the *afpyrG* marker at the 5'-end and inserted into the pESC-URA vector	This study
pJZ11	a 1026 bps PCR fragment of the upstream region from *pcr11010* from genomic DNA of *P. crustosum* PRB-2 was fused to 297 bps of the downstream region, a 1111 bps PCR fragment of two-thirds of the *afpyrG* marker at the 5'-end originated from pFK23 and inserted into the pESC-URA vector	This study
pJZ12	two-thirds of the *afpyrG* marker at the 3'-end (1103 bps) originated from pFK23 was fused to the 844 bps PCR fragment of the downstream region from *pcr11010* from genomic DNA of *P. crustosum* PRB-2 and inserted into the pESC-URA vector	This study
pJZ21	a 1929 bps PCR fragment of *pcr11010* with its terminator region (632 bps) from genomic DNA of *P. crustosum* PRB-2 were inserted into the pJN017 vector under the control of the *gpdA* promoter	This study

Table S3: Primers used in this study

Primer	Oligonucleotide sequence 5'-3'	Uses
5UTR_pESCf1	ATATACCTCTATACTTTAACGTCAAGGAGCTTCCTGATCCAGCTCGTAG	Amplification of the upstream region of *pcr4870* from *P. crustosum*
5UTR_300br1	GAGTATACAATAGCAAGATAATAGAAACACTGTGAAATGGGGCACCTTG	
300b_5UTRf1	TTTCAATATTCCAAGGTGCCCCATTTCACAGTGTTTCTATTATCTTGC	Amplification of 357 bps from downstream region of *pcr4870* from *P. crustosum*
300b_gpdAr1	gaccgtccgtctctccgcatgTATGGTAACCTGAAATATACTGAATTCC	
gdpApyrGf1	catgcggagagacggacg	Amplification of 2/3 from the *afpyrG* gene (5'end) for split marker cloning
pyrG_pESCr1	GGGCCCTATAGTGAGTCGTATTACGGATCcatcctccgaggctgaagac	
gdpApyrGr1	cggccgcattctgtctgagag	Amplification of 2/3 from the *afpyrG* gene (3'end) for split marker cloning
pyrG_pESCf1	TTAATATACCTCTATACTTTAACGTCAAGaacccgagaactcctggacc	
3UTR_pyrGf1	agtgcctcctctcagacagaatgcggccgCGTGTTTCTATTATCTTGC	Amplification of the downstream region of *pcr4870* from *P. crustosum*
3UTR_pESCr1	GGGCCCTATAGTGAGTCGTATTACGGATCCCGCAACGGCGACGAATATC	
5ku70ver_f1	GAATGTTAGCGGGTAGGATTG	Screening of Δ*pcr4870* transformants
pyrGver_r1	gctccatattctccgatgatg	
pyrGver_f2	gaggaaggctgcatacattgtgcc	
3ku70ver_r2	GGCGTTCTGTCTGGTATGTCTCG	
ku70ver_r2	CAAACTAGCAATTAATGTCCAAAGC	Screening of Δ*pcr4870* Δ*pyrG* transformants
ku70ver_f1	CAATATTCCAAGGTGCCCC	
5pyrG_f	gctagcgagagttattctgtgtctg	Amplification of 2/3 from the *afpyrG* gene (5'end) for split marker cloning
5pyrG_r	GAAATCAACTTCTGTTCCATGTCGACGCCCgaggctgaagacacatccg	
3pyrG_f	GATCCGTAATACGACTCACTATAGGGCCCgaactcctggacctcgctg	Amplification of 2/3 from the *afpyrG* gene (3'end) for split marker cloning
3pyrG_r	Gcggccgcattctgtctg	
traBup_f	GATCCGTAATACGACTCACTATAGGGCCCgtccaggagcacgaggtgag	Amplification of the upstream region of *pcr11010* from *P. crustosum*
traBup_r	cacccagagcctcatcactttgcatcgacgggcctttgatggcagg	
traB300_f	tcgatgcaaagtgatgag	Amplification of 297 bps from downstream region of *pcr11010* from *P. crustosum*
traB300_r	ttcgtcagacacagaataactctcgctagctctgactggaaattctgtg	
traBdown_f	gtgcctcctctcagacagaatgcggccgCtcgatgcaaagtgatgag	Amplification of the downstream region of *pcr11010* from *P. crustosum*
traBdown_r	AAATCAACTTCTGTTCCATGTCGACGCCCgttcgtggtcttaagttg	
pyrG_ver2	gagacaggccacatcggtgctgtattcctc	Screening of Δ*pcr11010* transformants
traBup_v_f2	gcaaggatagccctggaagtgccagtagag	
pyrGver1	ctattggacgcggtgccgactttatcatcg	
traBdo_v_r2	ctgcctcggtatctgggattggcttcattc	
traB_pJN17f	CTACCCCGCTTGAGCAGACATCACCGGCatggaagactttaaagttaag	Amplification of the *pcr11010* gene from *P. crustosum*
traB_pJN17r	CTCAACACCATATTTTAATCCCATGTGGGCgcggagcattacatcactc	
An_traB_5Vf	gatgctctcaagaccggcg	Screening of *A. nidulans traB* expression transformants
An_traB_5Vr	CCTCTCTAACCTCTGGTTCGC	
An_traB_GVf	ggctgagcggcacttctc	
An_traB_GVr	ctcatgcattcagcgagaggg	
An_traB_3Vf	CGAGTCtACTGAAGGTgGGCTTC	
An_traB_3Vr	gccaaggcaggaggtcaatg	

Table S4: ^{1}H NMR data of compound **1** and **2**

Compound	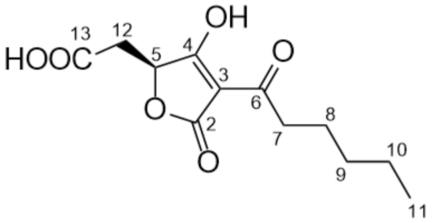	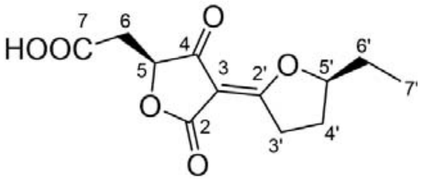
	Viridicatic acid (**1**, DMSO-d_6)	Crustosic acid (**2**, DMSO-d_6)
Position	δ_H, multi, *J* in Hz	δ_H, multi, *J* in Hz
5	4.39, dd, 9.2, 3.6, 1H	4.36, m, 1H
6	-	2.48, m, 1H
		2.33, m, 1H
7	2.61, m, 2H	-
8	1.44, m, 2H	-
9	1.24, m, 2H	-
10	1.24, m, 2H	-
11	0.85, t, 7.1, 3H	-
12	2.69, dd, 16.0, 3.5, 1H	-
	2.21, dd, 16.0, 9.2, 1H	
3‘	-	2.75, ddd, 15.4, 9.1, 6.6, 1H
		2.61, m, 1H
4‘	-	1.56, m, 1H
		1.36, m, 1H
5‘	-	4.47, m, 1H
6‘	-	1.45, m, 1H
		1.27, m, 1H
7‘	-	0.84, t, 3H
7-OH	-	8.51, s, 1H
13-OH	not detectable	-

The NMR data correspond very well to those reported previously (Fan et al. 2019; Fan et al. 2020).

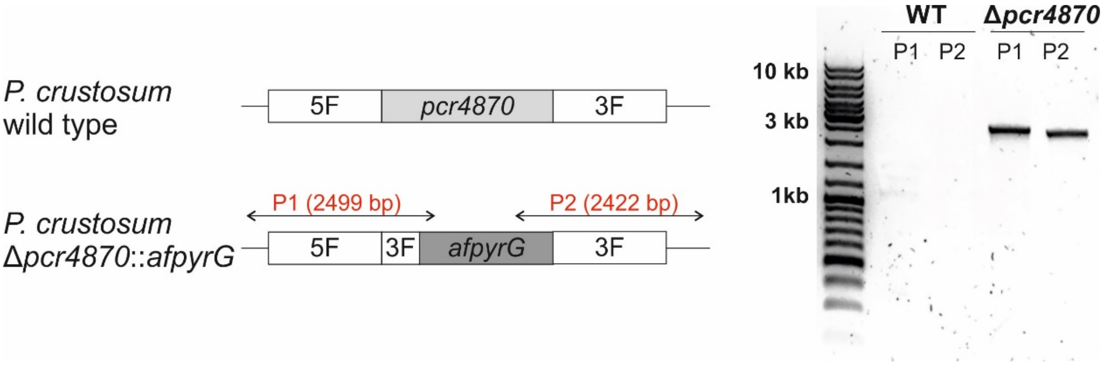

Fig. S1 PCR verification of Δ*pcr4870* mutant by amplification of two different fragments from genomic DNA of wild type and Δ*pcr4870* mutant (schematic representation)

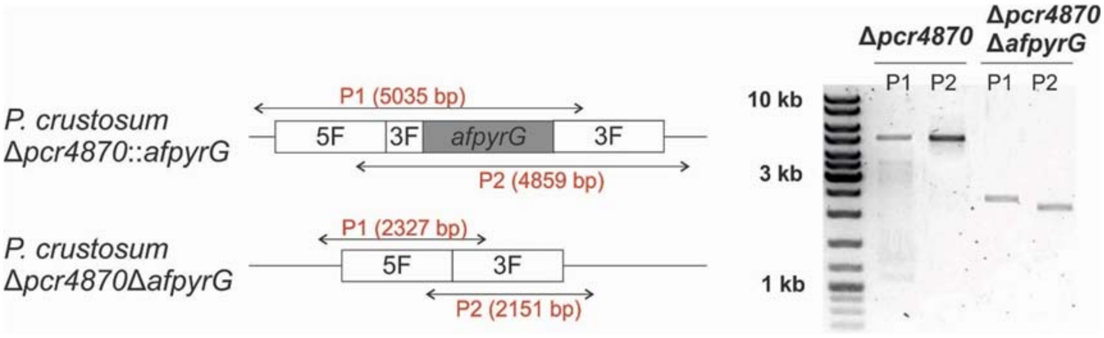

Fig. S2 PCR verification of Δ*pcr4870*Δ*afpyrG* mutant. Amplification from two different fragments from Δ*pcr4870*::*afpyrG* and Δ*pcr4870*Δ*afpyrG* strains proved the presence/absence of the *afpyrG* marker (schematic representation).

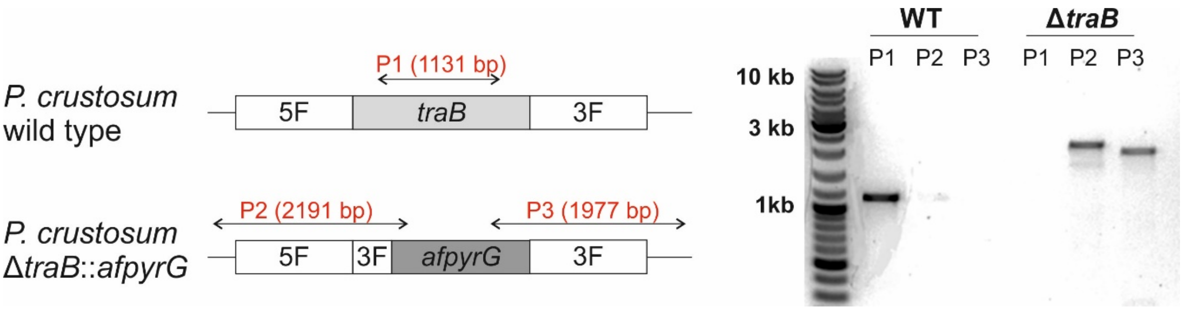

Fig. S3 PCR verification of Δ*traB* mutant by amplification of two different fragments from genomic DNA of wild type and Δ*traB* mutant (schematic representation)

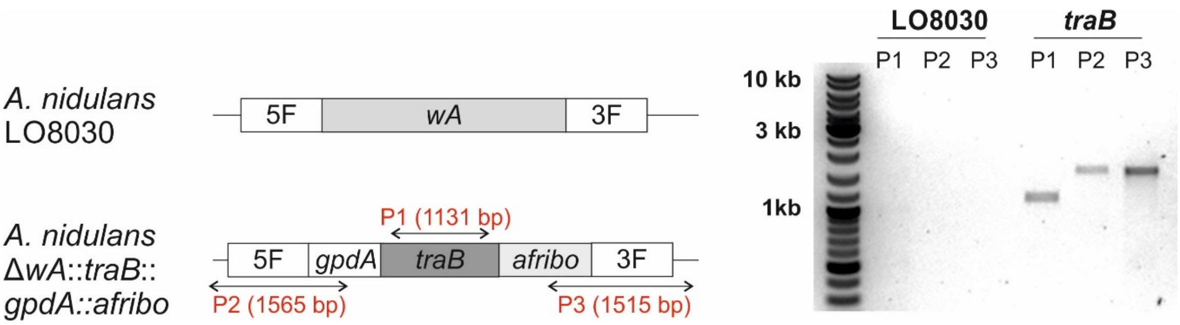

Fig. S4 PCR verification of *traB* overexpression mutant by amplification of three different fragments including the *traB* gene itself from genomic DNA of *A. nidulans* LO8030 and *traB* expression strain JZ01 (schematic representation)

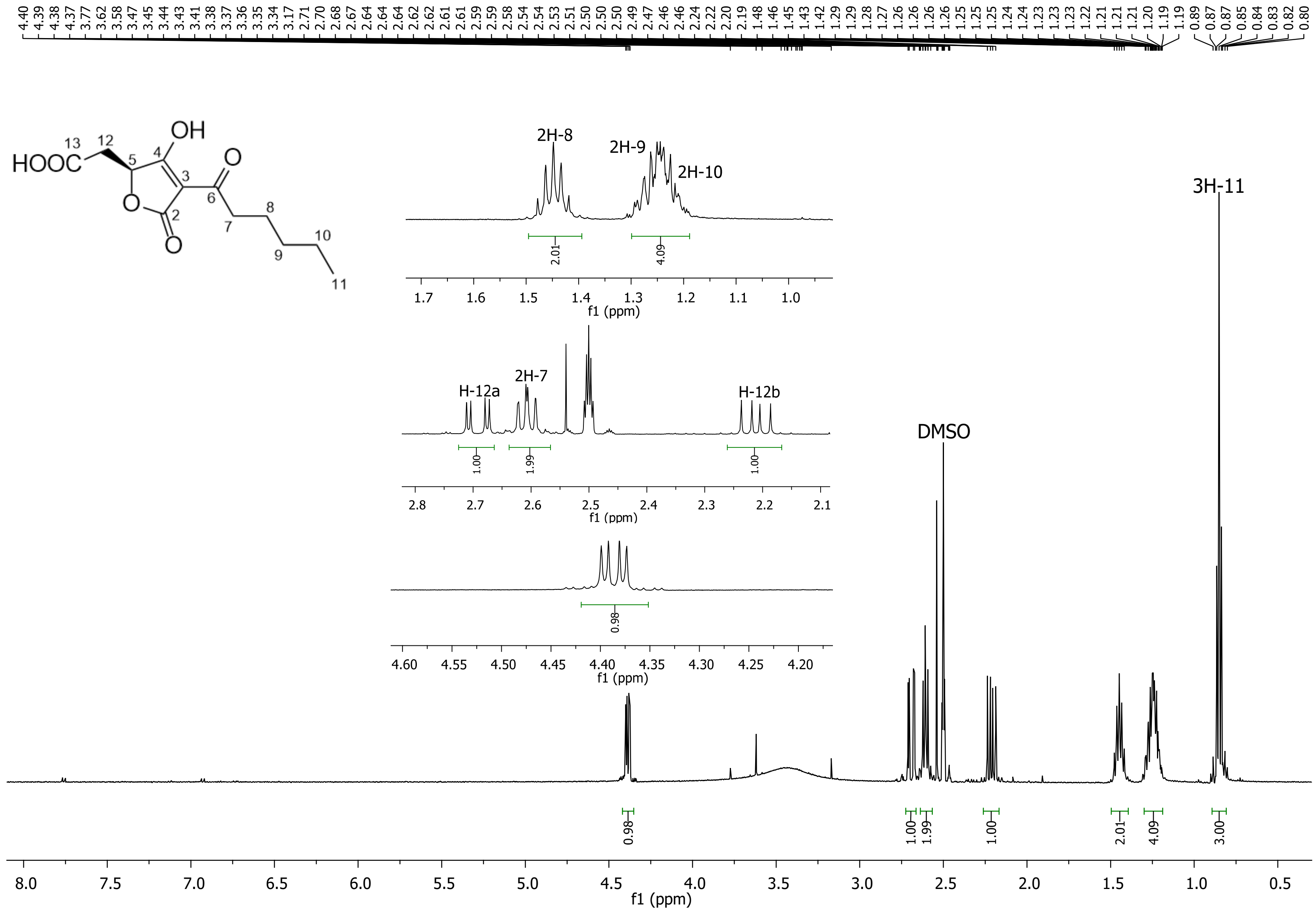

Fig. S5 ^{1}H NMR spectrum of compound **1** in DMSO-d6 (400 MHz)

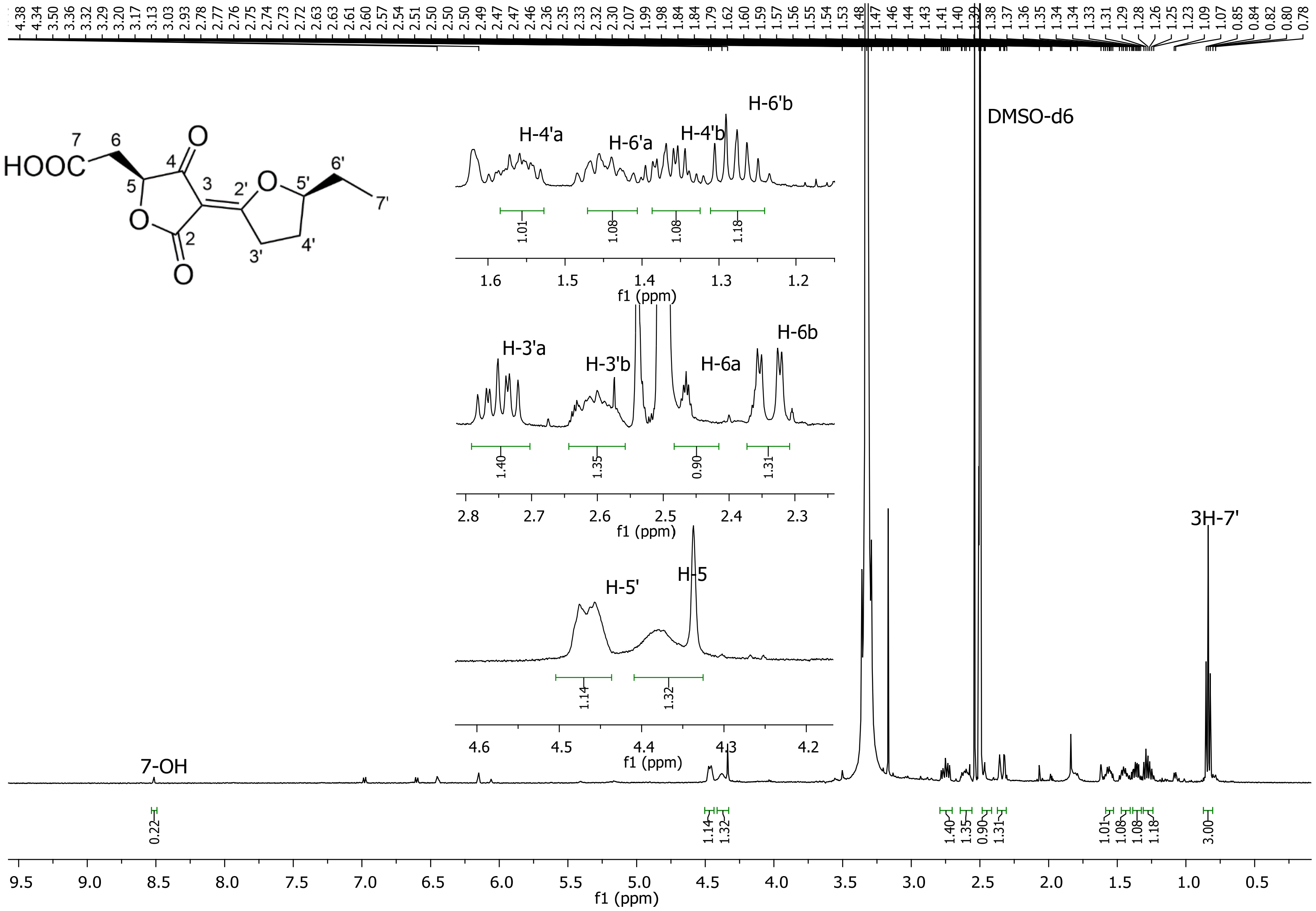

Fig. S6 ^{1}H NMR spectrum of compound **2** in DMSO-d$_6$ (400 MHz)

References

Chiang YM, Ahuja M, Oakley CE, Entwistle R, Asokan A, Zutz C, Wang CC, Oakley BR (2016) Development of genetic dereplication strains in *Aspergillus nidulans* results in the discovery of aspercryptin. Angew Chem Int Ed Engl 55:1662-1665.

Fan J, Liao G, Kindinger F, Ludwig-Radtke L, Yin W-B, Li S-M (2019) Peniphenone and penilactone formation in *Penicillium crustosum via* 1,4-Michael additions of *ortho*-quinone methide from hydroxyclavatol to γ-butyrolactones from crustosic acid. J Am Chem Soc 141:4225-4229.

Fan J, Liao G, Ludwig-Radtke L, Yin W-B, Li S-M (2020) Formation of terrestric acid in *Penicillium crustosum* requires redox-assisted decarboxylation and stereoisomerization. Org Lett 22:88-92.

Kindinger F, Nies J, Becker A, Zhu T, Li S-M (2019) Genomic locus of a *Penicillium crustosum* pigment as an integration site for secondary metabolite gene expression. ACS Chem Biol 14:1227-1234.

Wu G, Ma H, Zhu T, Li J, Gu Q, Li D (2012) Penilactones A and B, two novel polyketides from Antarctic deep-sea derived fungus *Penicillium crustosum* PRB-2. Tetrahedron 68:9745-9749.
